# Address on Oral Hygiene*Read before the National Dental Association October 23-26, 1917. Reprinted from *Journal N. D. A*.

**Published:** 1918-10

**Authors:** Haven Emerson

**Affiliations:** Commissioner of Health, New York City


### ADDRESS ON ORAL HYGIENE*

BY DR. HAVEN EMERSON, COMMISSIONER OF HEALTH, NEW YORK CITY

It is a particular pleasure to have the opportunity of expressing the gratitude of the Department of Health of New York City to the profession of dentistry which has assisted us in fulfilling, though not completely so far, our duty to the public, and particularly to the public school child.

Some years ago we tried to equip our medical forces with the necessary dental auxiliaries so that we could undertake preventive medicine through dental hygiene and treatment in the public schools. Finding the public was not sufficiently liberal to afford even the modest salaries we asked for dentists, we looked around to see what other assistance we could get. We thought of using nurses, and it was found we could not legally employ anybody to do even cleansing work on the teeth until the law had been changed. In co-operation with the representatives of the First and Second District Dental Societies here, it was agreed that an effort should be made to change the state law which permits the practice of dental hygiene by those who have not had

*Read before the National Dental Association October 23-26, 1917. Reprinted from *Journal N. D. A.*

the training of dentists. Before the Department of Health could employ any graduate nurses they were absorbed by the practicing dentists, and the department was unable to get any of those who had graduated for its use.

We are looking to the dental hygienists of the future to advance preventive dentistry as the visiting school nurse has advanced preventive medicine in the community.

I have yet to see a group of some hundreds of dentists appear before the Board of New York and demand proper consideration for their profession. I hope to see that in the future. We have asked that at least one dentist be given to the city of New York to take care of the defective teeth of the tuberculous patients whom we must send away to places where dentistry is inaccessible. Free dental care for the tuberculous poor is not provided for; you will not care for these patients in your private offices if you know these patients are tuberculous or if your other patients know they are. It is important to see that this fundamental equipment is provided, so that poor tubercular patients may get their teeth cared for in a scientific way to enable them to masticate and digest their food. We have not yet been able to get equipment in New York.

Furthermore, we have a large and increasing number of cases of syphilis in the communicable stage of this disease, and believe there must be official supervision for the care of these people, for their recovery will depend upon septic treatment, and proper care of the teeth, in order to restore them to health and usefulness, and you are well acquainted with the dangers that may result from infection with syphilis if patients are treated without a knowledge of the dentist. The dentist himself is running a great risk. It is impossible to look the problem of public health squarely in the face without recognizing the tremendous effect of syphilis in its communicable stage and the necessity of our combating it with every means at our disposal, and the syphilitic and tuberculous patients receive but scant consideration at the hands of the profession which should be prepared to treat their important dental defects.

It was not long ago that I attended a meeting of dentists in New York, and the question that was put to them was, shall we or shall we not assume the responsibilities as well as the privileges of a liberal profession? Shall we retrograde into a business or shall we advance to the full privileges of a profession? At that time it was necessary to take a very active part in the extension of the school dental clinics which are now one of the great assets of the public schools, and the stand the dentists took was worthy of the profession, and from that time on, at considerable personal sacrifices dentists have given a good deal of time at a nominal salary so that the children of the schools, who would otherwise be uncared for, should at least have a fair start in life.

Up to the age of two we have some control and supervision of the little babies of the city. We have direct supervision of 50,000 through the baby health stations. From the age of six to sixteen, they are all under constant supervision in the schools. From the age of two to the age of six children have not the benefit of medical protection. It is during that period that they develop most of the preventable defects which must be corrected when they enter school, and that is exactly the period of life when the community neglects dental care absolutely. If you talk to the mothers of these children as we have done in connection with our work, you will find they will say to you, ''Didn't suppose it was worth while to bother about the child's teeth because they are only temporary anyway, and they will soon be out.''

That is the attitude taken by mothers, and it must be changed by you. I should like to see the members of the National Dental Association see to it that teaching in this matter is immediate and persistent and widespread, so that preventive dentistry may begin where it belongs, with the first teeth. The first preventive care of the baby begins during the period of pregnancy of the mother. The pre-natal care of the expectant mother is where we do more good to the baby than any time subsequently in the child's life.

I want to give you a little idea of one of the other aspects of preventive work in New York City which has impressed us very much of late. We were notified that a fraudulent salvarsan was upon the market, a fraudulent imitation of the well known drug used in the treatment of syphilis. It was ordinary table salt with a little yellow dye. It could be put up in glass ampules, covered in an aluminum case, and put upon the market for less than fifty cents, and yet it was sold for five to ten dollars. We found where the product came from.

Two men were in charge, both of whom claimed to be physicians, and in the same establishment there was a diploma mill providing graduation diplomas to would-be physicians and dentists. We found the dies, the parchments, the finished articles, and they were being sold from $500 to $1,500 apiece, and widely circulated in this and adjoining cities. We have the men under bail and through the fortunate co-operation of the State Department of Education and the prosecuting attorney of the city, we shall succeed in getting these people safer than they are under bail. This was done by the activity of the dental and medical professions for their own protection, but as a mere accident in protecting the drug supply of New York by the Health Department.

The Department of Health of New York recognizes its responsibility to the children of the city to see that they get into proper hands, and it looks to the medical and dental professions to protect these children by rendering them suitable service.

There is one other aspect of the subject I want to speak about briefly, namely, that more food is lost and wasted through improper mastication than there is lost from careless housewives. I think as a profession you should direct your energies and thoughts towards food administration and conservation and call the people's attention to the fact that the more perfect the mastication of food is, the more food we will be able to send to our allies, and in this way you will do more for the country in your particular sphere than many people who rush around and put up signs on the walls of our buildings. I ask your co-operation in supporting the public health movement all through this country. I should like to see a personal appeal made to the Board of Estimate that dentists be paid adequate salaries for treating and looking after the 800,000 public school children, so that these children may not follow in the paths of their parents, but have a better chance for physical development and growth.

